# Enhancing corrosion resistance of biodegradable magnesium with dicalcium phosphate dihydrate and Chlorella sp. biomass

**DOI:** 10.1016/j.isci.2024.110761

**Published:** 2024-08-19

**Authors:** Lizeth del Carmen Gutierrez Pua, Lily Margareth Arrieta, Juan Carlos Rincon Montenegro, Leonardo Antonio Di Mare Pareja, Yaneth Pineda Triana, Ana Fonseca Reyes, Virginia Nathaly Paredes Mendez

**Affiliations:** 1 Department of Mechanical Engineering, Universidad del Norte, Km 5 Via Puerto Colombia, Barranquilla, Colombia; 2 Department of Metallurgical Engineering, Universidad Pedagogica y Tecnologica de Colombia, Avenida Central del Norte 39-115, Tunja, Boyacá, Colombia; 3 Mechanical Engineering Department, Universidad del Norte, Km5 Vía Puerto Colombia, Barranquilla, Colombia; 4 Biomedical Engineering Department, Universidad Simón Bolívar, Barranquilla, Colombia

**Keywords:** Biotechnology, Industrial biotechnology, Materials in biotechnology

## Abstract

Magnesium shows promise as a material for temporary fixation, yet its rapid corrosion poses health risks due to metal ion release. To mitigate these concerns, a biofunctionalization approach involving dicalcium phosphate dihydrate (DCPD) compounds and Chlorella sp. biomass was employed via electrodeposition, silanization, and dip-coating. Surface characterization using XRD, FTIR, and SEM confirmed successful deposition and immobilization. Corrosion behavior was assessed through electrochemical, immersion, and atomic absorption tests, revealing improved resistance and reduced Mg^2+^ ion release. The coatings demonstrated significant enhancement in corrosion resistance, guarding against pitting and cracks. The findings suggest the potential of Mg/DCPD and Mg/DCPD/microalgae coatings in addressing corrosion-related risks in temporary fixation applications, promising improved biocompatibility and longevity for medical implants.

## Highlights

• Mg biofunctionalization with DCPD and *Chlorella* sp. optimized for corrosion resistance
• *Chlorella* sp. immobilized in DCPD, depositing lipids, polysaccharides, and proteins
• DCPD/microalgae coatings cut Mg current density and corrosion, up to 61% efficiency
• Mg/DCPD and Mg/DCPD/microalgae coatings stopped corrosion-induced pitting and cracks

## Graphical abstract

**Figure:**
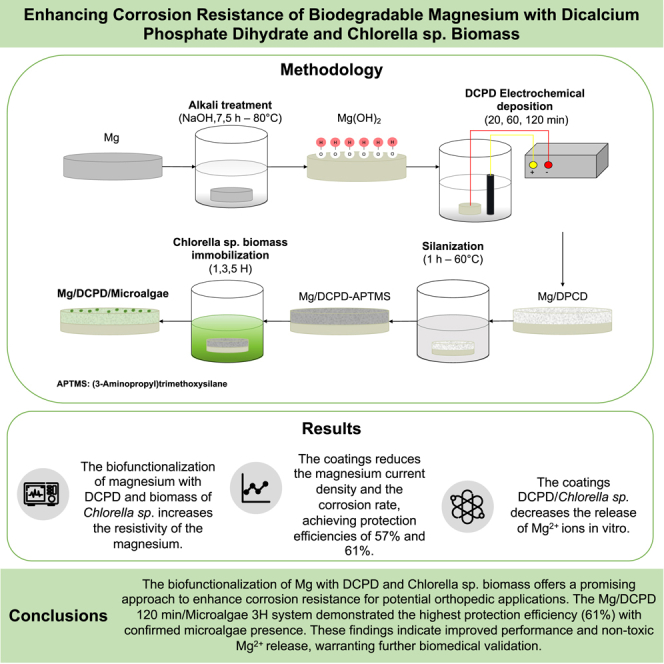

## Introduction

In recent decades, advancements in materials processing and medicine have led to increased utilization of metallic materials as temporary fixation devices. However, to mitigate risks such as corrosion and stress-shielding-induced bone weakening, their application necessitates removal after the bone tissue has healed.^1^ For this reason, biodegradable alloys have been studied as replacements for traditional materials. These alloys gradually degrade in the body, eliminating the need for a second surgical intervention to remove the implant and reducing associated health risks to the patient.^1^^,^^2^^,^^3^

Among biodegradable metallic materials, magnesium stands out as an excellent candidate for temporary implants due to its interaction with the body’s physiological fluids. It forms soluble and non-toxic products that can be safely eliminated through urine, posing minimal health risks. However, magnesium exhibits a high degradation rate in aqueous solutions, which can lead to complications such as osteolysis, mineral loss—especially calcium—bone degeneration and weakening, rapid mechanical degradation, formation of hydrogen bubbles, and tissue-implant gaps.^4^^,^^5^^,^^6^^,^^7^

Recently, various strategies have been investigated to control the degradation rate of magnesium, thus improving its performance and encouraging its use in temporary fixation elements.^5^^,^^8^^,^^9^ Among these, chemical surface modification has been extensively studied and remains a prominent technology for orthopedic implants due to the stable and strong bonds it promotes between the surface and the immobilized molecule, which can withstand the extreme conditions of the human body during and after implantation.^9^^,^^10^^,^^11^^,^^12^^,^^13^^,^^14^ In the context of biomedical applications, calcium phosphate (CaP) has garnered significant attention for surface modification. Coatings utilizing this ceramic are favored because they incorporate elements similar to those found in the mineralized inorganic phase of human bone. This similarity enhances biocompatibility and promotes favorable interactions between the implant and surrounding biological tissues.^15^^,^^16^ The release of Ca^2+^ and PO_4_^3−^ ions facilitates cell interaction on the implant surface, thereby promoting the growth of new bone tissue and providing greater *in vivo* stability over an extended period.^16^ Incorporating calcium phosphate (CaP) as a coating on magnesium alloys has demonstrated effectiveness in enhancing the corrosion resistance of magnesium. This improvement contributes to enhanced biocompatibility and osseointegration of the implant surface^6^^,^^15^^,^^17^^,^^18^^,^^19^^,^^20^ Moreover, these coatings serve to isolate the implant from direct contact with the physiological environment, facilitating surface mineralization and promoting osteogenic differentiation.^21^

Various phases of CaP have been utilized for coating orthopedic devices, including dicalcium phosphate dihydrate (DCPD) (CaHPO_4_ 2H_2_O), hydroxyapatite (HA) (Ca_10_(PO_4_)_6_ (OH)), tricalcium phosphate (whitlockite) (Ca_3_(PO_4_)_2_) and octacalcium phosphate (Ca_8_H_2_(PO_4_)_6_ 5H_2_O).^21^^,^^22^ DCPD, in particular, exhibits higher solubility compared to other CaP phases. It is relatively easy to deposit on a metallic substrate and serves as a precursor for the more stable hydroxyapatite (HA) phase, which mimics natural bone mineral and exhibits excellent biological activity.^21^^,^^22^^,^^23^ DCPD is recognized as an intermediate in biomineralization processes such as bone formation and dissolution of dental caries.^24^ Moreover, DCPD has been employed in metallic implants to enhance the concentration of calcium and phosphate ions at the tissue-implant interface, thereby promoting improved osseointegration of the biomaterial.^25^

In general, CaP coatings on magnesium (Mg) enhance corrosion resistance, biocompatibility, stimulate bone regeneration, and prolong the lifespan of implants. However, in physiological environments, aggressive ions like Cl-can infiltrate the Mg-CaP interface through pores or channels, compromising the coating’s effectiveness. To address this issue, researchers have explored the interaction of CaP with organic molecules to enhance coating performance.^6^^,^^23^ Studies have demonstrated that compounds such as EDTA,^26^ glucose^27^ and DNA^28^ can induce the formation of dense and refined CaP crystalline grains, thereby improving corrosion resistance and biocompatibility. Effective inhibitors typically incorporate nitrogen, sulfur, oxygen, phosphorus, and either a multiple bond or an aromatic ring. Some examples include plant extracts, chitosan, and cellulose, which possess structures or functional groups capable of acting as green corrosion inhibitors through biofunctionalization processes. Additionally, amino acids, known for their excellent biocompatibility, high purity, and cost-effectiveness, represent promising candidates for green inhibitors.^6^

Microalgae are unicellular eukaryotic organisms capable of photosynthesis, depending on water, sunlight, carbon dioxide, and various nutrients for their survival and growth.^29^^,^^30^ *Chlorella*, a species of ubiquitous microalgal, shows antibacterial,^31^^,^^32^ antioxidant,^31^^,^^32^^,^^33^ anti-inflammatory,^32^^,^^33^ antimicrobial,^32^ immunomodulatory,^33^ hemagglutination,^32^ and healing^32^^,^^34^ properties, and is highly biocompatible with the human body.^34^^,^^35^ It quickly absorbs and assimilates nutrients for growth as well. This species of microalgae contains vitamins E, B, and C, as well as minerals such as iron, magnesium, potassium, and calcium,^31^^,^^36^ making it suitable for pharmaceutical applications and tissue engineering.^32^^,^^34^^,^^35^^,^^37^^,^^38^^,^^39^ Several studies have shown various health benefits of Chlorella, including its β-1,3-glucan content, which can eliminate free radicals and reduce blood lipids.^40^^,^^41^ Additionally, it can increase hemoglobin concentration, lower blood sugar levels, and act as a hypocholesterolemic and hepatoprotective agent during malnutrition and ethionine poisoning.^41^ Specifically, *Chlorella* sp. contains peptides that could prevent cell damage and stimulate tissue generation.^35^ This microalga provides high-quality proteins with amino acid profiles that meet the standards established by the WHO/FAO/UNU for essential amino acids in human nutrition.^31^^,^^35^^,^^36^^,^^42^^,^^43^^,^^44^^,^^45^

The exceptional properties of *Chlorella* sp. position it as a promising candidate for enhancing the chemical biocompatibility and functionality of biomedical coatings, a central focus of this study. Consequently, our research aims to biofunctionalize magnesium elements with DCPD and *Chlorella* sp., optimizing the electrodeposition time of CaP and the immobilization time of microalgae biomass. The primary objective is to determine the system that exhibits superior corrosion resistance, as indicated by the resistivity conferred by the coating (Figure 13). This investigation aims to pave the way for potential applications of these biofunctionalized materials in orthopedic settings.

## Results and discussion

### Surface characterization

#### SEM

**Figure 1:**
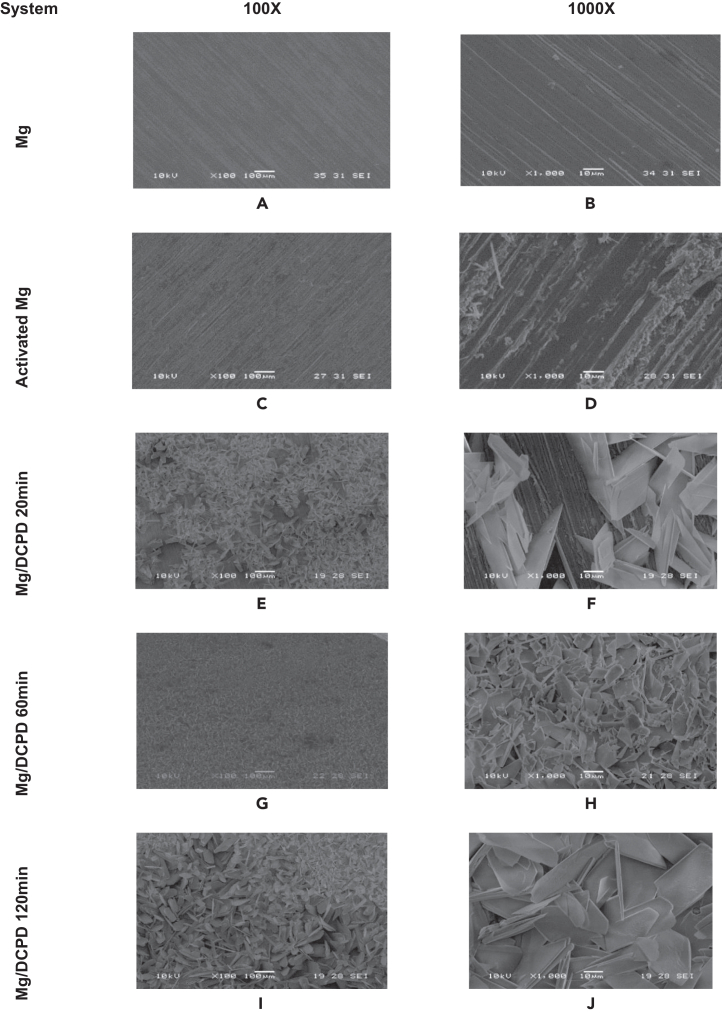
SEM morphologies for the indicated Mg/DCPD systems (A and B) Untreated Mg. (C and D) Activated Mg. (E and F) Mg/DCPD 20 min. (G and H) Mg/DCPD 60 min. (I and J) Mg/DCPD 120 min at 100X and 1000X.

Figure 1 illustrates the surface morphology of the untreated Mg alloy before and after DCPD electrochemical deposition (ED) across the three evaluated durations. In Figures 1A and 1B abrasive scratches from mechanical polishing are visible, but the surface appears evenly distributed. Upon activation of the substrate, large flakes oriented parallel to the polishing direction are evident, characteristic of Mg(OH)_2_, confirming successful activation^46^^,^^47^ (Figures 1C and 1D). Additionally, Mg(OH)_2_ crystals enhance surface preparation for subsequent film deposition by increasing hydroxyl groups, thereby improving interaction with surface-adhered molecules.^48^^,^^49^

The DCPD coating in Figures 1E and 1F consist of scale-shaped crystals randomly distributed on the surface. After 20 min of ED, uncovered sections indicate incomplete and uneven coating deposition, potentially compromising integrity. In the Mg/DCPD 60 min system (Figures 1G and 1H), a more homogeneous deposit is observed with improved crystal distribution and compact growth, enhancing coverage of the magnesium surface. Notably, after 120 min of ED, the Mg surface is fully covered by DCPD (Figures 1I and 1J), where scales grow in multiple directions with overlapping, resulting in a compact and uniform coating (Figure 1J). Highlights that DCPD crystal size increases with ED time, leading to a more integral and homogeneous coating.^50^ Overall, these images confirm successful deposition of the CaP compound on the Mg surface.

Considering the results obtained with the DCPD coating, an ED time of 120 min was selected due to its optimal coverage and uniformity. Following this, *Chlorella* sp. biomass will be immobilized on the DCPD-coated layer. Three immersion times in the microalgae solution will be evaluated: 1, 3, and 5 h.

**Figure 2:**
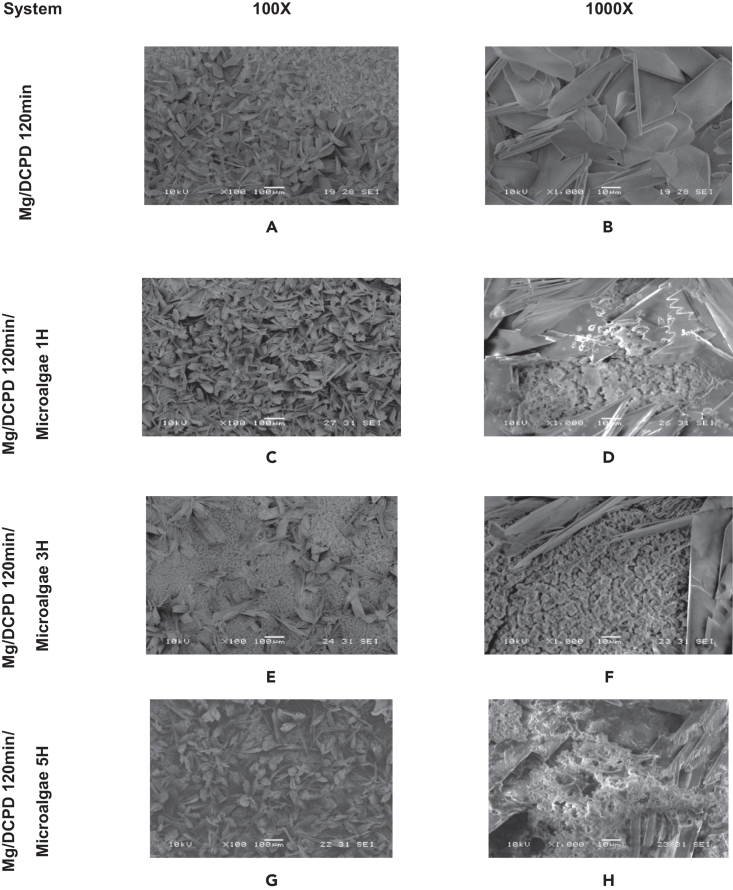
SEM morphologies of the indicated Mg/DCPD/Microalgae (A and B) Mg/DCPD 120 min. (C and D) Mg/DCPD1200 min/microalgae 1H. (E and F) Mg/DCPD120 min/microalgae 3H. (G and H) Mg/DCPD120 min/microalgae 5H at 100X and 1000X.

**Figure 3:**
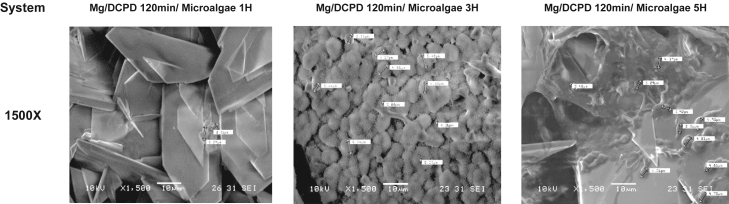
Radius of Chlorella sp. for the indicated Mg/DCPD/Microalgae

Figures 2 and 3 present the morphology of the surface after the immobilization process of the microalgae on the DCPD coating for the three immersion times evaluated. After immersion in the microalgae solution for 1-h, small clumps of biomass were identified among the DCPD crystals. However, the images show a predominance of the CaP morphology, indicating that this immersion time resulted in only slight coverage of the sample (Figures 2C and 2D).

In the Mg/DCPD 120 min/microalgae 3H system, an increase in the amount of immobilized biomass was observed. Figure 2E shows that, although DCPD crystals are still visible, the substrate is mostly covered by microalgae. *Chlorella* sp. lodged among the DCPD crystals, creating large aggregates and even completely covering some crystals (Figures 2E and 2F).

Finally, after 5 h of immersion, a decrease in the amount of biomass on the surface was observed compared to the 3-h sample. The distribution of the coating was similar to the Mg/DCPD 120 min/Microalgae 3H system, but with a greater presence of DCPD crystals. The biomass clumped between the crystals and covered portions of them (Figures 2G and 2H).

According to the literature, cells of the *Chlorella* sp. species have a rounded and aggregated geometry with a diameter ranging from 3 to 8 μm.^51^^,^^52^^,^^53^^,^^54^ For all evaluated systems, this information was verified. Figure 3 shows the diameters of some microalgae cells identified in each coating. All values were within the range reported by other authors. Additionally, the spherical geometry of the cells was observed. These images confirm the successful immobilization of the *Chlorella* sp. biomass on the DCPD coating.

#### X-ray diffraction

**Figure 4:**
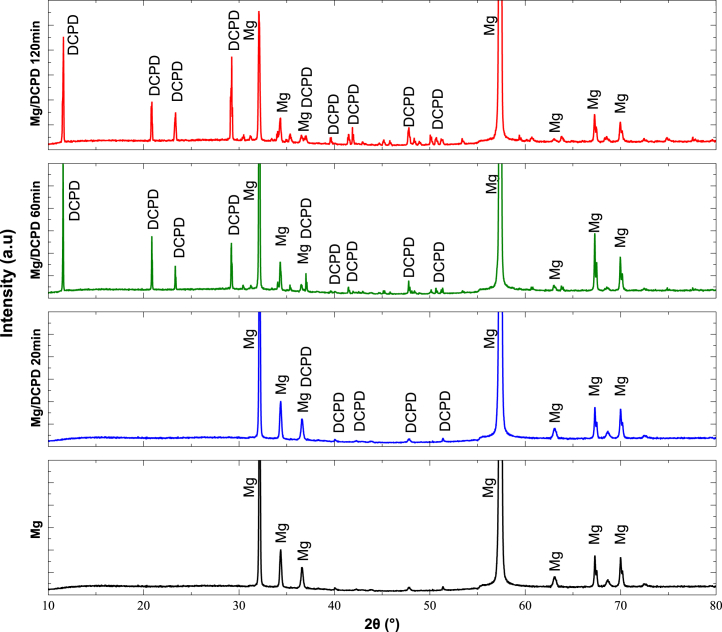
XRD patterns for DCPD coatings

Verification of the presence of DCPD on magnesium was performed using X-ray diffraction (XRD). Figure 4 shows the patterns obtained. The main diffraction peaks of Mg were identified in the untreated sample, which are also clearly observed in the treated substrates, indicating that the coatings are thin. They are composed of DCPD crystals, and some coincidences with hydroxyapatite peaks were detected, which could be due to monoclinic or amorphous crystalline structures.^55^ These results confirm the presence of DCPD on the surface after the ED process for all evaluated times at approximately 2θ equal to 12°, 21°, 23.5°, 29°, 37°, 48°, and 51°, consistent with previous reports in the literature.^15^^,^^56^ With increasing ED time, the appearance of new hydroxyapatite peaks was observed, indicating a more uniform deposition of the coating on the surface and a greater presence of DCPD on the substrate.

#### Fourier transform infrared spectroscopy

**Figure 5:**
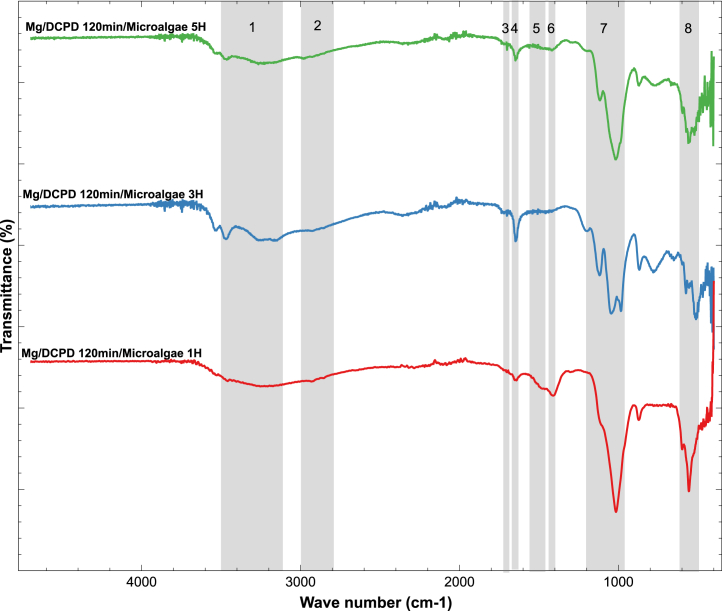
FTIR spectra for indicated systems

The identification of functional groups in the microalgae through FTIR is presented in Figure 5 and Table 1 shows the assignment of the spectral bands with their corresponding biomolecular functional group and origin. Eight regions were identified, which were correlated with the presence of macromolecules in *Chlorella* sp. biomass previously reported in the literature. The FTIR spectra show the presence of hydroxyl groups (OH), carboxyl groups (COOH), amines (NH_2_), and other groups associated with organic compounds. The peaks in the high-intensity region (1) at 3500-3100 cm^−1^ were attributed to the presence of OH groups linked to hydrogen, a characteristic structure of polysaccharides.^57^ In all samples, lipid regions (2 and 3) were identified in the range of 3000–2800 cm^−1^ and 1750–1700 cm^−1^. The peaks in this zone were attributed to the presence of C-H stretching vibrations and esters, ketones, and carboxylic acids (C=O), although a weak signal was observed in this region, indicating a low presence of compounds containing functional groups associated with lipids.^57^ In the region (4, 5, 6, and 8), spectra related to the presence of proteins were identified.^57^^,^^58^^,^^59^ The functional groups associated with carbohydrates exhibit the strongest absorption between 1200 and 1000 cm^−1^, and the peaks identified in the range of 980–∼1200 cm^−1^ (7) can be related to the presence of polysaccharides.^59^

**Table 1:** Band assignment—FTIR

Peak	Wave range (cm^−1^)	Mg/DCPD 120 min/Microalgae 1H (cm^−1^)	Mg/DCPD 120 min/Microalgae 3H (cm^−1^)	Mg/DCPD 120 min/Microalgae 5H (cm^−1^)	Functional group	Biomolecular origin	Reference
1	3500–3100	3462,65	3470,29	3466,36	O-H stretching with hydrogen bonds	Polysaccharides	Gonzalez-Torres et al.^57^
		3221,01	3272,08	3241,37			
			3154,58				
2	3000–2800	2929,59	2919,95	2997,8	Aliphatic C-H Stretch	Mainly lipid	Gonzalez-Torres et al.,^57^ Ponnuswamy et al.,^59^ Giordano et al.^60^
		2858,52	2858,16	2920,3			
3	1750–1700	–	1726	1721,4	Ester, Ketone, and Carboxylic Acid (C=O) Stretch	Lipids, Chlorophyll, Carotenoid Pigments	Gonzalez-Torres et al.^57^
				1700,68			
4	1660–1610	–	1642,83	1648,18	C=O stretch	Protein (Amide I)	Gonzalez-Torres et al.^57^
5	1481–1585	1547,83	–	–	Amide C-N Stretch and N-H bend	Protein (Amide II)	Gonzalez-Torres et al.,^57^ Ponnuswamy et al.^59^
		1483,19					
6	1440–1395	1409,26	–	1420,33	Asymmetric flexion of CH_3_	Protein	Zeroual et al.,^58^ Ponnuswamy et al.^59^
7	980–∼1200	1015,34	1204,26	1219,62	C-*O*-C Stretch	Polysaccharides	Ponnuswamy et al.,^59^ Giordano et al.^60^
			1117,84	1117,84			
			1044,98	1016,05			
			983,91				
8	610–535	597,85	–	–	C-N-C	Protein	Bayramoǧlu et al.^61^
		559,06					

The organic compounds identified by means of FTIR correspond to those found in the composition of the microalgae biomass, therefore, these results confirm the successful immobilization of *Chlorella* sp. on DCPD.Electrochemical measurements.

#### Mg/DCPD coatings

Based on ISO 10993-15:2001standard,^62^ to obtain the biological evaluation of medical devices, the identification and quantification of degradation products must be carried out. Therefore, electrochemical impedance spectroscopy (EIS) was used to evaluate the corrosion behavior of the samples before and after treatment with DCPD and DCPD/microalgae.

**Figure 6:**
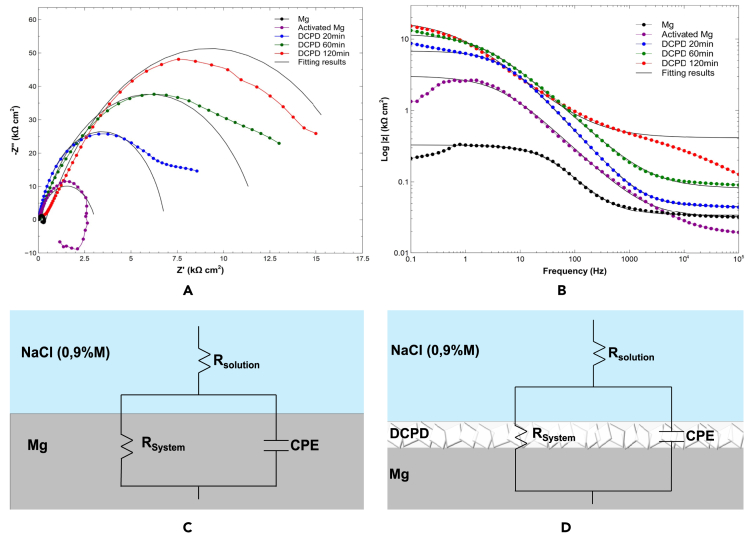
Electrochemical characterization of Mg/DCPD systems (A) Nyquist Plot. (B) Bode Plot of Log |z|. (C) Equivalent Circuit of Untreated Mg. (D) Equivalent Circuit of Mg/DCPD System.

Figure 6 displays the Nyquist and Bode plots of the DCPD systems. The Nyquist plots show a single capacitive loop with a larger diameter than the Mg substrate (with and without activation), indicating the presence of the coating by reducing charge transfer processes. Additionally, it is evident that the diameter of the DCPD loops increases as the electrodeposition time increases, confirming the formation of a compact coating with longer treatment times, as observed in the morphological analysis (Figure 1).

The Bode plot (Figure 6B) shows the variation of the impedance modulus with respect to frequency for the DCPD-electrolyte interface. The impedance modulus has higher values compared to the Mg substrate, once again demonstrating the presence of the phosphate coating and the inhibition of corrosion reactions. Considering that the impedance modulus depends not only on the thickness but also on the uniformity of the coating, it can be concluded that the coating deposited at 120 min of electrodeposition is the most uniform.^63^

For fitting the EIS data, the proposed and presented equivalent electrical circuits (EECs) are shown in Figures 6C and 6D, where R_solution_ represents the solution resistance, and R_system_ is the resistance of the coating. Considering the constant variation of the system over time resulting from surface inhomogeneity, coating variation, surface composition, and current and potential distributions induced by geometry, a constant phase element (CPE) will be introduced in the implemented circuits.^64^ Table 2 presents the values extracted from the EEC for each system, where n is the ideality coefficient. The R_system_ for the uncoated Mg was 17.6700 kΩ cm^2^, while for the DCPD coatings, the values were 6.778 kΩ cm^2^, 11.880 kΩ cm^2^, 17.670 kΩ cm^2^ for 20, 60, and 120 min, respectively. It is accepted that higher R_system_ values indicate higher corrosion resistance. Considering the results obtained from the Nyquist and Bode plots, it can be determined that the system that provides the highest corrosion resistance is Mg/DCPD 120 min.

**Table 2:** Fitting results for the electrochemical parameters of EIS

Sample	R_solution_	R_system_	CPE	*n*
	$k\Omega \;cm^{2}$	$k\Omega \;cm^{2}$	$k\Omega^{-1}cm^{-2}s^{n}$	
Mg	0.03389	0.29540	4,31E-08	0,8383
Activated Mg	0.03176	3.030	2,78E-08	0,7506
Mg/DCPD 20 min	0.04456	6.778	8,43E-09	0,8443
Mg/DCPD 60 min	0.04005	11.880	1,22E-08	0,7222
Mg/DCPD 120 min	0.41070	17.670	2,14E-08	0,6705

#### Statistical analysis of corrosion behavior of Mg/DCPD

A one-way ANOVA was conducted to determine if there is a significant difference in the corrosion resistance of Mg after DCPD treatments. The results indicate, with 95% confidence, that the electrodeposition time of DCPD on magnesium influences the material’s resistivity (*p* value = 1.93E^−11^ < 0.05). Furthermore, a pairwise mean comparison of treatments was performed using Fisher’s least significant difference (LSD) method to determine the minimum difference required between the means of two electrodeposition times to be considered statistically different, considering a confidence level of α = 0.05. The LSD results are shown in Table 3.

**Table 3:** LSD for DCPD coating

LSD DCPD electrodeposition time	0.72914		Resistance levels	
Systems	Mean difference	Conclusion	Mg	Low resistance
Mg - DCPD 20 min	6.364	SIGN	DCPD 20 min	Low-Medium resistance
DCPD 20 min—DCPD 60 min	5.276	SIGN	DCPD 60 min	Medium resistance
DCPD 60 min—DCPD 120 min	5.880	SIGN	DCPD 120 min	High resistance

All evaluated mean pairs differ significantly, and the 120-min electrodeposition time yields significantly higher resistivity than the other treatments. Four resistance levels were identified, where untreated Mg offers the lowest corrosion resistance, and the DCPD coating at 120 min provides the highest resistivity. These results are consistent with those obtained in the electrochemical tests and confirm the successful electrodeposition process.

#### Mg/DCPD/microalgae

**Figure 7:**
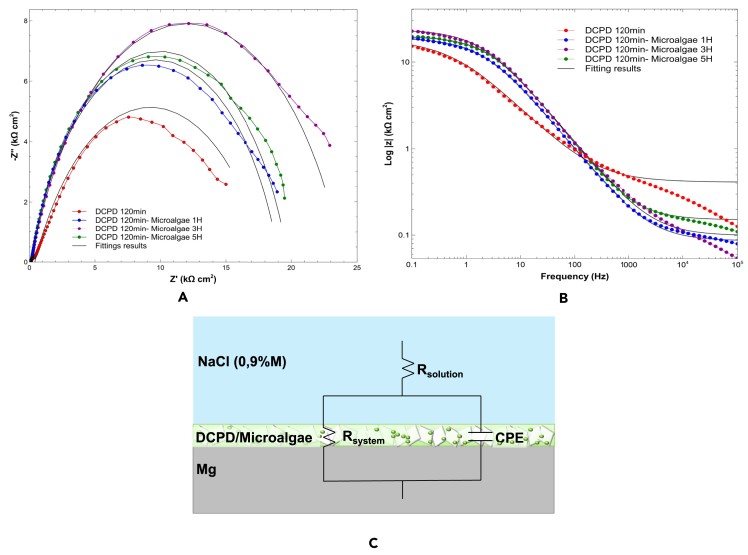
Electrochemical Characterization of Mg/DCPD/microalgae system (A) Nyquist plot. (B) Bode Plots of Log |z|. (C) Equivalent circuits.

For the Mg/DCPD/microalgae coatings, Figure 7 shows the Nyquist diagrams, Bode plots, and polarization curves of the evaluated systems. The Nyquist diagrams for all the coatings with Chlorella sp. exhibit high-frequency capacitive loops, which can be attributed to charge transfer and corrosion products formed on the surface.^15^^,^^55^ No low-frequency inductive loops were identified, indicating that the coating remained intact and provided protection to the substrate. Figure 7A shows that the immobilization of microalgae in DCPD caused an increase in the capacitive loop diameter for all three immobilization time spans compared to Mg/DCPD 120 min. The 1-h and 5-h times exhibited similar behavior, while the 3-h span showed the largest capacitive loop, implying that this system provides greater corrosion resistance.

On the other hand, the Bode plot (Figure 7B) shows a slight increase in impedance for the three immobilization spans of the DCPD system with microalgae compared to DCPD 120 min, which confirms the analysis of microalgae surface coverage. Among the systems with microalgae, very close |Z| values are observed, with the highest impedance value obtained at 3 h, indicating that the coatings provided similar but superior corrosion resistance to the Mg/DCPD 120 min system.

Similar to the DCPD coating evaluation, fitting of the graphs was performed using EECs (EEC) with CPE (Figure 7C). The values extracted from the ECC for each system are presented in Table 4. The R_system_ for Mg/DCPD 120 was determined to be 17.670 kΩ cm^2^ as determined in the previous section. For the systems with microalgae, R_system_ values of 18.990 kΩ cm^2^, 23.670 kΩ cm^2^, and 19.620 kΩ cm^2^ were obtained for 1 h, 3 h, and 5 h, respectively. It is estimated that higher R_system_ values correspond to greater corrosion resistances. Therefore, these results indicate that the system that provides the highest corrosion resistance is Mg/DCPD 120 min/microalgae 3H.

**Table 4:** Fitting results for the electrochemical parameters of EIS

System	R_solution_	R_system_	CPE	*n*
	$k\Omega \;cm^{2}$	$k\Omega \;cm^{2}$	$k\Omega^{-1}cm^{-2}s^{n}$	
Mg/DCPD 120min	0.4107	17.67	2,14E-08	0,6705
Mg/DCPD 120 min/Microalgae 1H	0.1208	18.99	6,06E-09	0,7832
Mg/DCPD 120 min/Microalgae 3H	0.0989	23.67	6,87E-09	0,7407
Mg/DCPD 120 min/Microalgae 5H	0.1509	19.62	5,54E-09	0,7881

#### Statistical analysis of corrosion behavior of Mg/DCPD/microalgae

A one-way ANOVA was performed to determine if immersion in *Chlorella* sp. biomass significantly influences the corrosion resistance provided by the phosphate coating. The results indicate, with a 95% confidence level, that the immersion time in microalgae significantly affects the resistivity offered by the DCPD 120 min coating and, therefore, provides greater corrosion resistance to Mg (*p* value = 8,01E^−04^ < 0.05). The evaluation of pairwise treatment means using Fisher’s LSD method indicated no significant difference between the DCPD 120 min coating and DCPD 120 min/microalgae 1H (Table 5). This implies that statistically equal results will be obtained when immersing the samples in microalgae for 1 h or working with the Mg/DCPD 120 min system without microalgae. This may be due to the low presence of biomass observed in the system after 1 h of immersion. Coatings, which predominantly exhibits DCPD morphology, generated similar resistivities (Figures 2A and 2C). The rest of the pairwise comparisons showed a significant difference in their means. In particular, the Mg/DCPD 120 min/microalgae 3H system offers statistically significant and different results compared to the other evaluated immersion time spans and the DCPD coating. Three levels of resistance were identified. DCPD 120 min and microalgae 1H systems have lower resistance compared to the DCPD 120 min/microalgae 3H coatings.

**Table 5:** LSD for DCPD/Microalgae coating

LSD immersion time span in microalgae	±2.949		Resistance levels	
Systems	Mean difference	Conclusion	DCPD 120 min	Low resistance
DCPD 120 min – DCPD 120 min/Microalgae 1H	0.690	NO SIGN	DCPD 120 min/Microalgae 1H	
DCPD 120 min/Microalgae 1H– DCPD 120 min/Microalgae 3H	6.213	SIGN	DCPD 120 min/Microalgae 5H	Medium resistance
DCPD 120 min/Microalgae 3H– DCPD 120 min/Microalgae 5H	−3.853	SIGN	DCPD 120 min/Microalgae 3H	High resistance

#### Polarization curves

**Figure 8:**
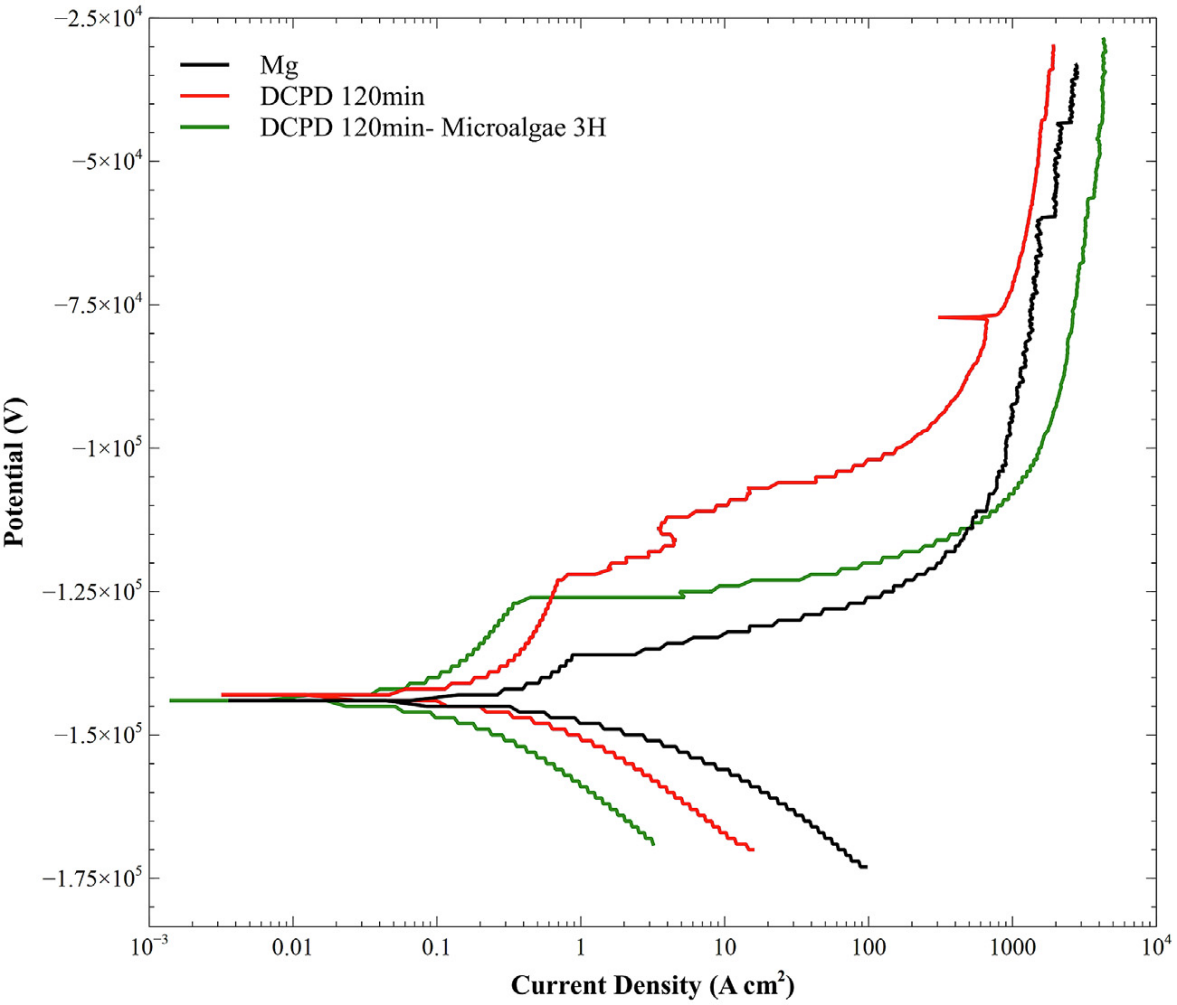
Polarization curves

Figure 8 shows the polarization curves for the coatings that provide high corrosion resistance, and Table 6 presents the fit results. The corrosion potential (E_corr_) did not show a difference between the untreated sample and the DCPD and microalgae coatings. However, the corrosion current density (I_corr_) significantly decreased for the coatings. The DCPD 120 min/microalgae 3H system exhibited the lowest I_corr_. A lower current density indicates better corrosion behavior for the sample.^65^^,^^66^ This is consistent with the results obtained for the corrosion rate. The presence of CaP compounds and the microalgae significantly reduced the corrosion rate of Mg. The coating efficiencies were 57% and 61%, respectively. These results indicate that the coatings provide significant corrosion protection to magnesium.

**Table 6:** Electrochemical measurements derived from the polarization curves

Sample	b_A_ (V/decade)	b_C_ (V/decade)	I_corr_ (mA)	E_corr_ (V)	Corrosion Rate (mm/year)	%η
Mg	138,8E-3	105,4E-3	3,830	−1,440	0.1740662	–
DCPD 120 min	79,60E-3	131,7E-3	1,640	−1,420	0.0746506	57%
DCPD 120 min/Microalgae 3H	475,5E-3	152,3E-3	1,490	−1,440	0.0678688	61%

#### Immersion test

**Figure 9:**
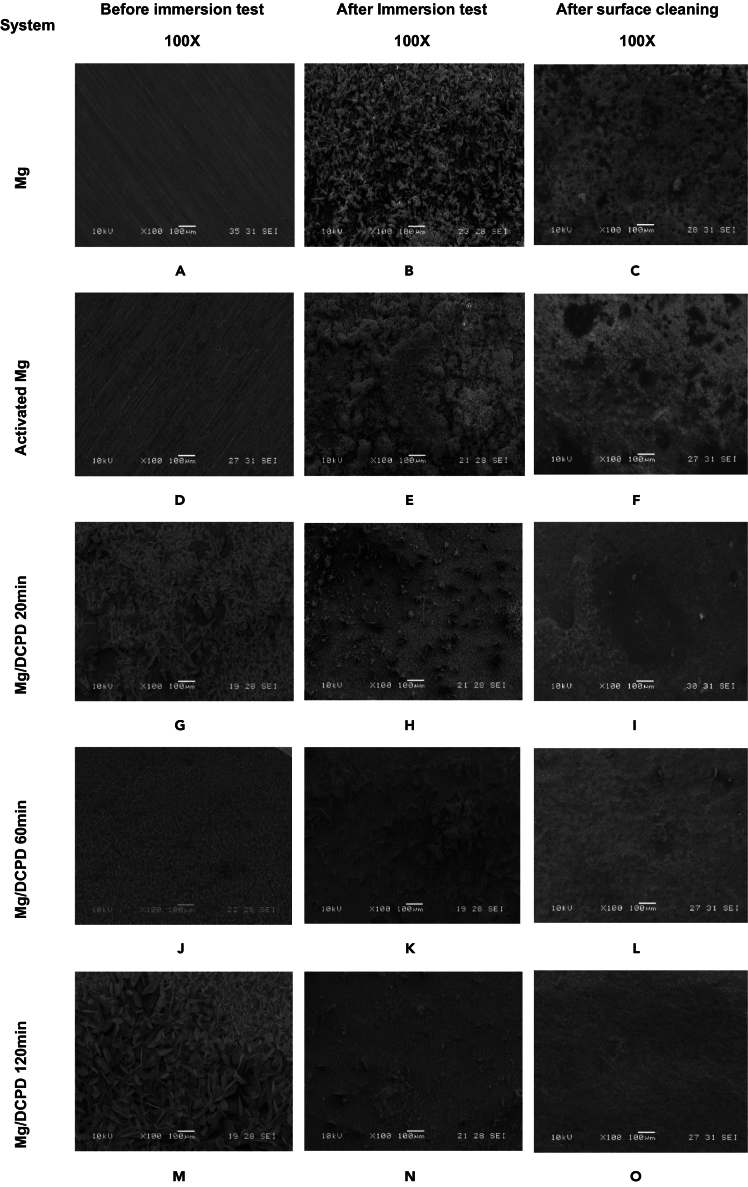
Surface morphology before the immersion test, after the immersion test and subsequent cleaning for the indicated Mg/DCPD systems (A-C) Untreated Mg. (D-F) Activated Mg. (G-I) Mg/DCPD 20 min. (J-L) Mg/DCPD 60 min. (M-O) Mg/DCPD 120 min for Before and After Inmersion Test, and After Surface Cleaning at 100X.

**Figure 10:**
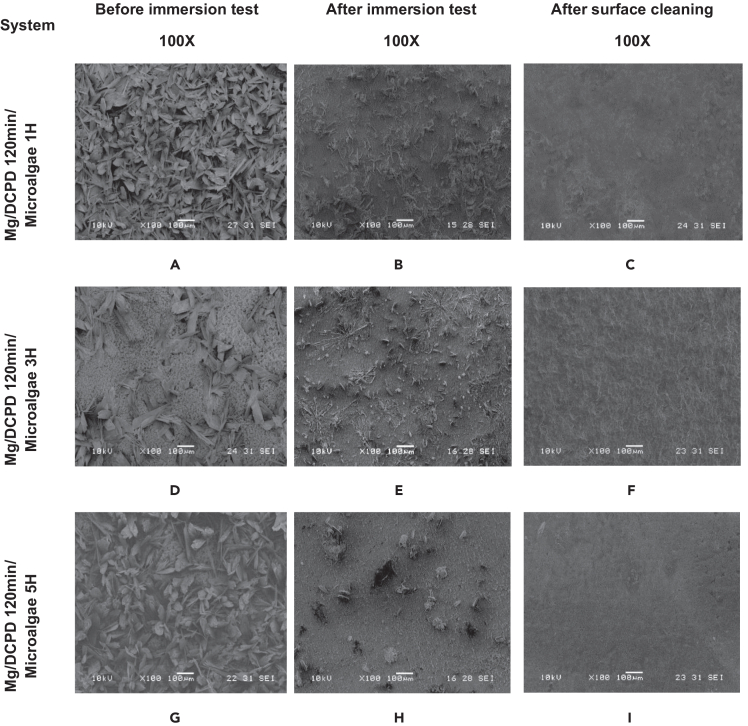
Surface morphology before the immersion test, after the immersion test and subsequent cleaning for the indicated Mg/DCPD/Microalgae systems (A-C) Mg/DCPD120min/microalgae 1H. (C-F) Mg/DCPD120 min/microalgae 3H. (E-H) Mg, DCPD120 min/microalgae 5H for Before and After Inmersion Test and After Surface Cleaning at 100X.

Figures 9 and 10 present the morphology of the Mg/DCPD and Mg/DCPD/microalgae systems before and after the immersion test.

For the untreated Mg/DCPD sample, irregular corrosion products are observed after the test and before the cleaning process (Figures 9B and 9C). After removing the deposits, severe pitting corrosion and cracking are evident (Figure 9C). In the pretreated specimen, a layer of mineralization composed of prism-shaped crystals covers the surface, indicating an increase in corrosion products (Figure 9E). After cleaning, cracks are present on the surface, though not as pronounced as on bare Mg, and shallow cavities are visible (Figure 9F).

In the Mg/DCPD systems, new products appear on the surface, likely associated with the evolution of corrosion reactions. As electrodeposition (ED) time increases, the presence of these deposits decreases. The Mg/DCPD 120 min coating shows a more uniform morphology with fewer particles compared to the 20 and 60-min ED times (Figures 9H–9K and 9N). After cleaning, the surface exhibits pitting but is more preserved compared to the other evaluated samples (Figures 9I, 9L, and 9O).

In samples with microalgae, a similar morphology to the CaP systems is observed. Before removing the corrosion products, particle presence on the surface decreases as the immobilization time increases (Figures 10B–10E and 10H). After cleaning, the Mg/DCPD 120 min/microalgae 1H sample exhibits a morphology similar to the DCPD 120 min system; however, no pits or cavities are observed on the substrate (Figure 10C). After 3 h of immersion, a rough-looking surface is observed without cavities or pits (Figure 10G). Finally, after 5 h of immersion in microalgae, a surface with a more homogeneous appearance is obtained compared to the 3-h immersion sample, although pitting is observed (Figure 10I).

None of the systems showed the presence of microalgae or DCPD crystals post-cleanup. However, the DCPD/Microalgae coatings provided significant protection to Mg, reducing pitting, cavities, and corrosion cracks. After cleaning, the Mg/DCPD 120 min/microalgae 3H sample exhibited a more preserved morphology without significant macroscopic damage.

**Figure 11:**
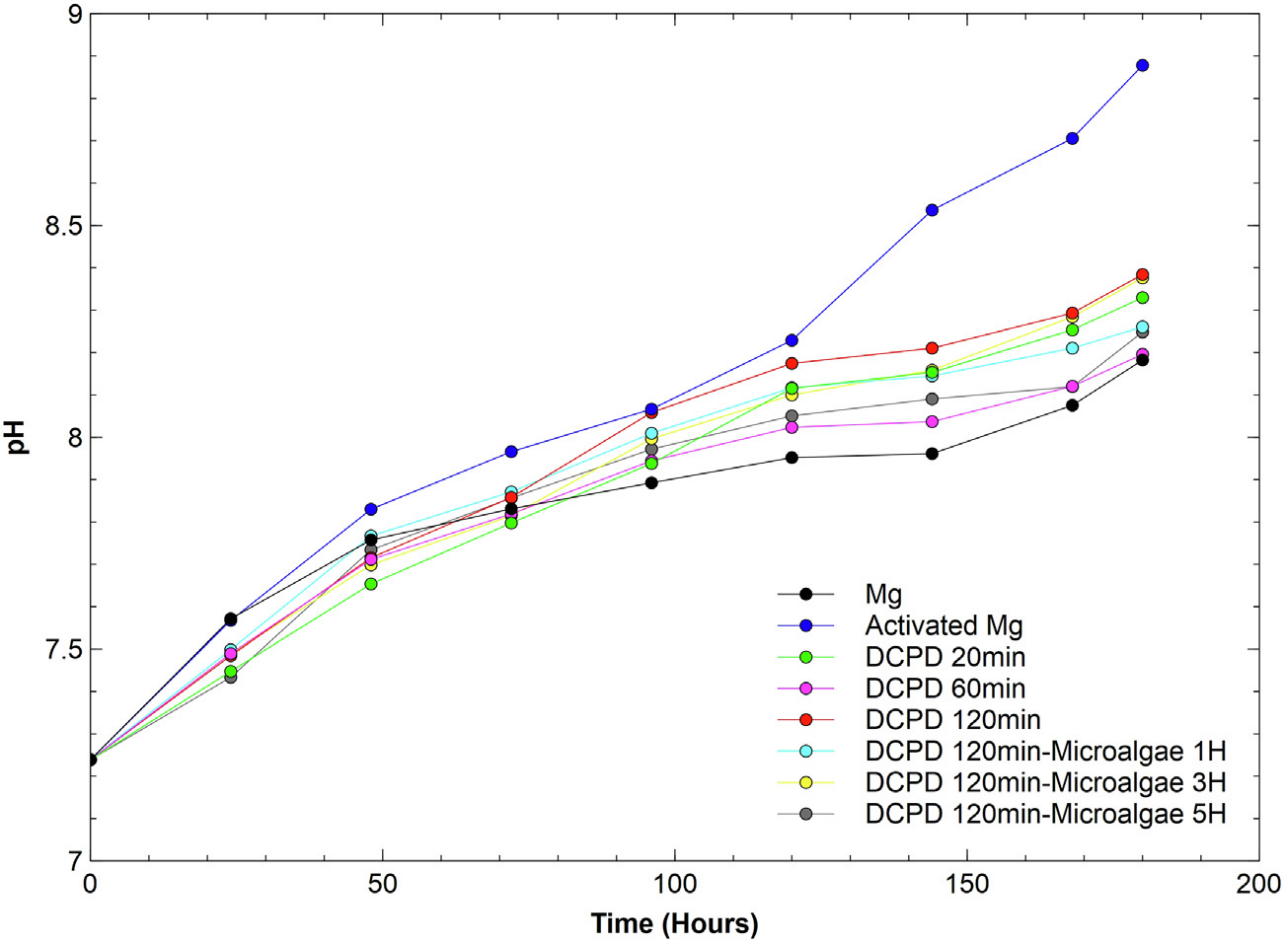
pH graph of the immersion test

The pH values of the samples during the 180-h immersion test showed a similar behavior pattern with a gradual increase in an alkaline range (Figure 11). Among the DCPD and microalgae coatings, the Mg/DCPD 120 min and Mg/DCPD 120 min/microalgae 3H systems exhibited the highest pH values at 8.4. However, all systems produced a higher pH compared to untreated Mg after 100 h, which could be attributed to the formation of a layer of corrosions layer or material passivation that provides temporary protection to the substrate and leads to a decrease in the corrosion of the sample. However, the activated Mg, which has a previously passivated surface, showed a more pronounced pH increase compared to the other specimens after 100 h of immersion, reaching values of 8.8 at the end of the test. In Figure 11, it can be seen that the pH values of the bare Mg starting from 140 h: 7.96 (144 h), 8.07 (168 h), and 8.18 (180 h), are very close to those presented by the Mg activated for more than 70 h, 7.96 (72 h), 8.7 (96 h), and 8.2 (120 h), which indicates a trend in the long-term behavior of the passivation layer. Furthermore, in the final phase of the test, the pH curve for Mg shows a more pronounced increasing trend compared to the other systems evaluated. This, together with the information mentioned previously, suggests that other strategies should be employed to improve the long-term corrosion protection of a previously passivated magnesium surface.

The results obtained are consistent with what is reported in the literature. The rapid corrosion of Mg leads to local alkalization, which can induce an imbalance in physiological reactions *in vivo* and even cause alkaline poisoning with pH values higher than 7.8.^67^^,^^68^ This indicates that although the proposed coatings improve the corrosion resistance of Mg, as indicated in the previous tests, it is still necessary to evaluate other strategies to enhance this result.

**Figure 12:**
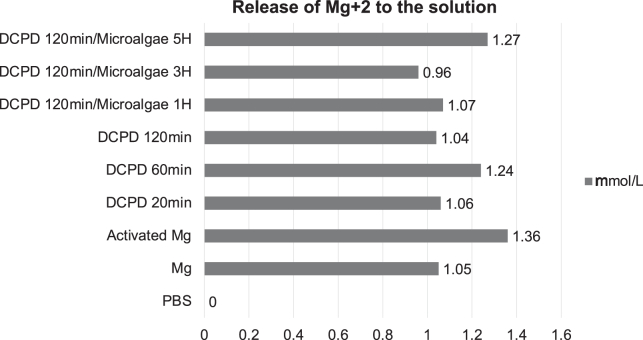
Mg2+ concentration after immersion test

In Figure 12, the AAS results are shown in terms of Mg^2+^ ion concentration in mmol/L. The normal contents of these ions in the blood are estimated to be between 0.70 and 1.10 mmol/L, and concentrations below 1.05 mmol/L are considered non-toxic.^69^^,^^70^ Mg/DCPD 120 min/microalgae 3H showed the lowest release of Mg^2+^ ions, followed by Mg/DCPD 120 min. These two results are consistent with those observed in the EIS test and the corrosion rate calculation, confirming the effect of these two coatings on the corrosion behavior of Mg.

**Figure 13:**
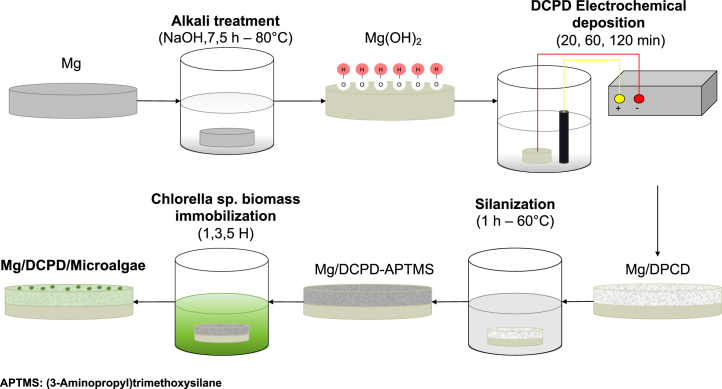
Preparation of the DCPD and DCPD/Microalga coatings on an Mg alloy

However, the other systems presented concentrations higher than 1.05 mmol/L, which means that although they can improve the corrosion behavior of Mg, they are not enough to reduce the ion release rate and could induce health complications of the patient. The activated sample exhibited the highest concentration of ions, which confirms the assumption about the rapid corrosion of the material and correlates with a pH of 8.9 during immersion, where the increase in pH of the medium is a result of the corrosion of the substrate.

### Conclusions

This study presented the biofunctionalization process of Mg with DCPD and *Chlorella* sp. biomass by optimizing the electrodeposition time of DCPD and the immobilization time of the microalgal biomass to identify the system that provides the best behavior against corrosion and guide its application as an orthopedic material. Based on the results obtained, the following conclusions can be drawn.

• The morphological analysis revealed the presence of DCPD crystals on the surface of the material for the three evaluated times. A homogeneous layer formed on the surface after 120 min, and the DCPD crystals exhibited improved distribution and closer growth, resulting in a uniform appearance. Furthermore, characterization of the Mg/DCPD coating by XRD confirmed that the deposited crystals corresponded to DCPD.
• The Nyquist plot showed an increase in capacitive diameter loop after the coatings compared to untreated Mg, with the increase being more pronounced with longer deposition times. Similarly, the Bode plots indicated that the Mg/DCPD 120 min coating was the most uniform, and the highest resistivity was achieved in the Mg/DCPD 120 min system.
• Statistical analysis indicated that, at the 95% confidence level, the DCPD deposition time influenced the Mg resistivity, and the LSD test showed that the 120-min electrodeposition time resulted in a significantly higher resistivity than the other treatments. Based on these results, the 120-min electrodeposition time was selected as the substrate for immobilization of the microalga, *Chlorella* sp.
• The morphological analysis revealed the presence of microalgae in the DCPD coating. The 3 and 5-h immersion spans showed a higher proportion of microalgae, *Chlorella* sp., lodged between the DCPD crystals, forming large congregations, and even covering some of them. FTIR was used to identify the organic compounds deposited on the surface, showing the presence of lipids, polysaccharides, and protein structures, which are characteristic compounds of microalgae biomass.
• The Nyquist plot showed an increase in the capacitive loop diameter after immersion in microalgae compared to the Mg/DCPD 120 min system. The Bode plots showed that among the systems with microalgae, similar |Z| values were observed; with the highest impedance value achieved after a 3 h microalga immobilization process.
• Statistical analysis indicated that, with a 95% confidence level, the immersion time in microalgae influenced the resistivity of Mg/DCPD. Similarly, the LSD test indicated that the DCPD 120 min/microalgae 3H system differed significantly from the other systems and resulted in the highest average resistivity.
• The polarization curves showed that the DCPD 120 min and DCPD 120 min/microalgae 3H coatings reduced the magnesium current density and the corrosion rate, achieving protection efficiencies of 57% and 61%, respectively.
• It is evident that the DCPD/microalgae coatings provided high protection to Mg, reducing the occurrence of corrosion induced pitting, cavities, and cracks after immersion tests. After cleanup, the Mg/DCPD 120 min/microalgae 3H system exhibited a more preserved morphology without significant macroscopic damage.
• The Mg/DCPD 120 min and Mg/DCPD 120 min/microalgae 3H systems exhibited a release of Mg^2+^ ions below 1.05 mmol/L, a value within the accepted range in blood (0.70–1.10 mmol/L), indicating that they are not chemically toxic.

Considering the results obtained in the three phases, it can be concluded that the biomass of *Chlorella* sp. and DCPD improve the corrosion behavior of Mg, and the properties of these materials could be beneficial in orthopedic applications. Specifically, the DCPD 120 min/microalgae 3H system offers better coating performance and greater corrosion resistance to Mg. However, it is advisable to evaluate strategies to lower the pH in future work, as the pH curves exhibited a similar behavior in all the samples, showing an upward trend in the alkaline range at pH between 8 and 9. These pH values could potentially lead to health complications in patients or alkaline poisoning *in vivo*.

In addition, future research should focus on analyzing protein fixation using chemical methods to develop a multilayer approach that enhances both the resistivity and chemical biocompatibility of the coatings. Further studies should include cell adhesion analysis to evaluate the biocompatibility of the DCPD/microalgae system, explore broader ED parameter ranges, examine the impact of cultivation conditions on microalgae performance, and investigate other microalgae species for their potential in biomedical applications.

### Limitations of the study

Despite the promising findings, several limitations remain in this study. These limitations provide avenues for future research to further enhance the understanding and performance of the biofunctionalized coatings. The results presented in this work are limited to the influence of the electrodeposition (ED) parameters for DCPD and the immobilization time of *Chlorella* sp. biomass. Future research should explore broader or different parameter ranges to better understand their effects on coating performance.

While surface characterization was performed, further in-depth analysis is needed. Techniques such as X-ray photoelectron spectroscopy (XPS) could provide both qualitative and quantitative data on the coating’s composition and the chemical state of the elements on the magnesium (Mg) surface. Additionally, the corrosion products formed on the samples after immersion tests were not fully analyzed. Using energy-dispersive X-ray spectroscopy (EDS) to identify the elements on the Mg surface under physiological conditions would help determine the nature of the immersion products and their impact on the coating’s biocompatibility.

To better understand the effect of *Chlorella* sp. on the biocompatibility of the coating, cell adhesion studies should be conducted. These studies would evaluate the DCPD/microalgae system’s performance in terms of cell growth and survival. Furthermore, the influence of cultivation conditions on the microalgae’s performance was not examined. Future studies should investigate how varying these conditions might affect the biofunctionalized coating’s results.

Given the potential of microalgae as corrosion inhibitors in biomedical applications, examining other species could yield valuable insights. Comparing the performance of different microalgae species in biofunctionalization processes would help identify the most effective organisms for enhancing metallic surfaces.

## Resource availability

### Lead contact

Further information and requests for resources and reagents should be directed to and will be fulfilled by the lead contact, Lizeth Gutierrez Pua (gutierrezdl@uninorte.edu.co, lizgutierrezpua@gmail.com).

### Materials availability

This study did not generate new unique reagents.

### Data and code availability

• All data reported in this paper will be shared by the lead contact upon request.
• This paper does not report original code.
• Any additional information required to reanalyze the data reported in this paper is available from the lead contact upon request.

## Acknowledgments

The authors acknowledge the support given by the Biotechnology laboratory of Universidad del Norte.

The authors acknowledge the financing of the: Ministry of Science, Technology, and Innovation of Colombia through the “Créditos educativos condonables para la formación de capital humano de alto nivel para las regiones”—Atlantico, Colombia. National master's modality. Call no. 809 of 2018.

## Author contributions

L.D.C.G.P.: investigation, validation, conceptualization, methodology, formal analysis, writing—original draft, visualization, figure design, writing—review and editing. L.M.A.P.: investigation, validation, conceptualization, methodology, writing—review and editing. J.C.R.M.: investigation, validation, conceptualization, methodology, writing—review and editing. L.A.D.M.P.: investigation, resources, conceptualization, writing—review and editing. Y.P.T.: investigation, resources, conceptualization, writing—review and editing. A.M.R.F.: investigation, conceptualization, formal analysis, writing—review and editing, supervision. V.N.P.M.: investigation, conceptualization, formal analysis, writing—review and editing, supervision. All authors have read and agreed to the published version of the manuscript.

## Declaration of interests

The authors declare no competing interests.

## STAR★Methods

### Key resources table

**Table:** 

REAGENT or RESOURCE	SOURCE	IDENTIFIER
**Chemicals, peptides, and recombinant proteins**		

Calcium nitrate tetrahydrate	Sigma-Aldrich	MFCD00149604, CAS: 13477-34-4
Ammonium dihydrogen phosphate	Sigma-Aldrich	MFCD00003396, CAS: 7722-76-1
Sodium nitrate	Sigma-Aldrich	MFCD00011119, CAS: 7631-99-4
3-aminopropyl trimethoxysilane	Sigma-Aldrich	MFCD00008206, CAS: 13822-56-5

### Experimental model

*Chlorella* sp. was obtained from the Biotechnology Laboratory at the Universidad del Norte (Barranquilla, Colombia), and cultured in Bold's Basal Medium (BBM).^71^ For culture preparation, 2 mL of Chlorella sp. were used in 148 mL of medium under constant agitation. The culture was maintained at a temperature of 20 ± 2°C, with a light intensity of 1760 lumens and a photoperiod of 12 h of light and 12 h of darkness. The pH of the culture medium was adjusted to 7.4. For the biofunctionalization process, it is crucial that the microalgae biomass has a high protein content. Therefore, *Chlorella* sp. was harvested during the exponential phase of the culture growth curve, reaching an absorbance of 1.70, which corresponds to approximately 9.99E6 cells/mL. This absorbance value was measured using a spectrophotometer, and the absorbance and culture parameters determined based on data previously studied and determined from preliminary tests conducted by the research group.

### Method details

#### Materials

Mg alloy (2.24 wt % Al, 97.76 wt % Mg) test pieces, with a diameter of 8 mm and a height of 20 mm, were used as the substrate. The samples were polished with SiC paper with grain sizes between 100 and 2000. Subsequently, they were washed in 96% ethanol for 5 min and dried with compressed air. Figure 13 shows the experimental procedure.

#### DCPD coating preparation

Before depositing DCPD, an alkaline pretreatment was performed using sodium hydroxide (NaOH) to generate a magnesium hydroxide (Mg(OH)_2_) layer.^72^ Hydroxyl groups in the oxide film on metals participate in the formation of chemical bonds between the surface of the material and the immobilized molecules. Additionally, they improve the resistance to corrosion and the biocompatibility of the substrates.^73^ Previous studies indicate that treatments with NaOH can increase the concentration of hydroxyl groups on the magnesium surface.^23^^,^^74^^,^^75^^,^^76^^,^^77^^,^^78^^,^^79^ Therefore, the previously polished specimens were immersed in a 3M NaOH solution for 7.5 h at 80 ± 5°C. Finally, they were rinsed in 96% ethanol for 30 s and dried with compressed air.

The coating was obtained through electrochemical deposition (ED). The electrolyte for CaP coatings contained a 0.042 M solution of calcium nitrate tetrahydrate (Ca(NO_3_)_2_ ·4H_2_O), 0.025 M of ammonium dihydrogen phosphate (NH_4_H_2_PO_4_) and 0.1 mol/L of sodium nitrate (NaNO_3_).^50^ The pH of the solution was set at 4.5, which allowed the formation of dicalcium phosphate dihydrate (DCPD) (CaHPO_4_ · 2H_2_O). The parameters of the electrodeposition process were established by Li et al..^50^ In short, the electrodeposition was carried out at a voltage of 2.5V, with a graphite plate as the anode and the pre-treated magnesium surface as the cathode. Three working times were evaluated: 20, 60, and 120 min, in order to determine the influence of the time variable on the corrosion resistance of magnesium.

#### Silanization

From the obtained DCPD coating, a silanization process was performed using (3-aminopropyl) trimethoxysilane (APTMS) at a concentration of 10 mM (432 μL of APTMS in 239.56 mL of 96% ethanol). The Mg/DCPD samples were immersed in the APTMS solution for 1 h at 60 ± 5°C. After the allotted time, the samples were rinsed with 96% ethanol for 30 s and dried with hot air for 3 min.

#### Immobilization of the biomass of Chlorella sp

For biomass immobilization, the samples were immersed in the microalgae solution at room temperature. Three immersion times were evaluated: 1, 3, and 5 h, in order to determine the influence of immersion time on the corrosion resistance provided by the system. The resulting coating was named to as Mg/DCPD/Microalgae.

#### Surface characterization

The morphology of the substrate surface was observed using a scanning electron microscope (SEM, JEOL model 5600). X-ray diffraction (XRD, Bruker, D8 Advance Family) was employed to verify the presence of the calcium phosphate compound on Mg using Cu Kα radiation at 25 mA and 40 kV. The measurement was performed in the range of θ=[10°−80°] with a scanning speed of 4°/min. Fourier-transform infrared spectroscopy (FTIR, Shimadzu with ATR detector) was used to identify the functional groups of the microalgae in the DCPD.

#### Electrochemical test

The corrosion behavior of the designed systems was evaluated using electrochemical impedance spectroscopy (EIS) with a GAMRY-ref. 600 Potentiostat/Galvanostat/ZRA. For this purpose, a setup with a conventional three-electrode cell configuration was considered: a silver/silver chloride (Ag/AgCl) electrode as the reference electrode, a platinum wire as the counter electrode, and the treated Mg as the working electrode (0.503 cm^2^). The electrolyte used was 0.9% NaCl. Before the measurements, the samples were immersed in the electrolyte for 10 min at 28°C to achieve steady-state conditions. Nyquist and Bode plots were recorded with a perturbing potential of 10 mV/ms over a frequency range of 0.1–1 × 10^5^ Hz. The tests were repeated three times.

#### Polarization curves

Corrosion parameters such as corrosion potential ($E_{corr}$) and corrosion current density ($i_{corr}$) were obtained using the Tafel extrapolation method. The samples that provided the highest resistance were selected, and the efficiency of the coating compared to untreated magnesium was evaluated. For this, the following equation was employed^80^^,^^81^^,^^82^:

$$\%\eta =\frac{\left({\text{CR}}_{\text{wt}}\right)-\;\left({\text{CR}}_{t}\right)}{{\text{CR}}_{\text{wt}}}.100\%$$

Where ${CR}_{wt}$ and ${CR}_{t}$ are the corrosion rates of the sample with and without the Coating. The tests were repeated three times.

#### Inmersion tests

An immersion test was carried out according to ASTM G31^83^ and BS EN ISO 10993-15:2001^62^ standards for the different systems: Mg, Mg/DCPD, and Mg/DCPD/Microalgae in phosphate-buffered saline (PBS) solution for 7 days at 37°C ± 0.5°C. The pH of the solution was adjusted to 7.4^84^ and pH control was maintained throughout the test. At the end of the test, the samples were cleaned with acetone in an ultrasound bath for 7 min. A soft-bristle brush was used to remove any remaining corrosion products. After the immersion test, 50 mL of the remaining electrolyte (PBS) for each system evaluated was analyzed using Atomic Absorption Spectroscopy (AAS, Shimadzu 7000, following SM 3111B: Direct Air Acetylene Flame Method) to identify and quantify the ions released in the solution. A qualitative analysis was performed to determine the system that exhibited the best performance in physiological environments in terms of biocompatibility and magnesium ion concentration.

### Quantification and statistical analysis

#### Evaluation of the results from DCPD electrodeposition on Mg

Based on the results from electrochemical impedance spectroscopy (EIS), a statistical analysis was performed using one-way analysis of variance (ANOVA) to determine if there is a significant difference in the corrosion resistance of Mg due to the electrodeposition (ED) process. Subsequently, if the ANOVA results indicated a significant difference, Fisher’s least significant difference (LSD) test was employed to identify the ED time span that provided the best performance, indicated by the highest resistivity. Both tests were conducted with a confidence level of 95% (α = 0.05).

#### Evaluation of the results from the biofunctionalization of microalgae on Mg/DCPD

Similarly, for the biofunctionalization phase, EIS results were analyzed using one-way ANOVA to determine if there was a significant difference in the corrosion resistance of Mg/DCPD due to the immobilization process of Chlorella sp. biomass. If the results from ANOVA showed a significant difference, Fisher’s LSD test was utilized to identify the immobilization time span that provided the best performance, again based on the highest resistivity. The confidence level for both tests was maintained at 95% (α = 0.05).
